# N-Acetyl-cysteine causes analgesia by reinforcing the endogenous activation of type-2 metabotropic glutamate receptors

**DOI:** 10.1186/1744-8069-8-77

**Published:** 2012-10-23

**Authors:** Matteo Bernabucci, Serena Notartomaso, Cristina Zappulla, Francesco Fazio, Milena Cannella, Marta Motolese, Giuseppe Battaglia, Valeria Bruno, Roberto Gradini, Ferdinando Nicoletti

**Affiliations:** 1 I.R.C.C.S. Neuromed, Pozzilli, Italy; 2 Department of Physiology and Pharmacology, University “Sapienza”, Piazzale Aldo Moro, 5, Rome, 00185, Italy; 3 Department of Experimental Medicine, University “Sapienza”, Rome, Italy

**Keywords:** Cystine/glutamate antiporter, N-acetyl-cysteine, mGlu2 receptors, Analgesia, AGS3

## Abstract

**Background:**

Pharmacological activation of type-2 metabotropic glutamate receptors (mGlu2 receptors) causes analgesia in experimental models of inflammatory and neuropathic pain. Presynaptic mGlu2 receptors are activated by the glutamate released from astrocytes by means of the cystine/glutamate antiporter (System x_c_^-^ or Sx_c_^-^). We examined the analgesic activity of the Sx_c_^-^ activator, N-acetyl-cysteine (NAC), in mice developing inflammatory or neuropathic pain.

**Results:**

A single injection of NAC (100 mg/kg, i.p.) reduced nocifensive behavior in the second phase of the formalin test. NAC-induced analgesia was abrogated by the Sx_c_^-^ inhibitor, sulphasalazine (8 mg/kg, i.p.) or by the mGlu2/3 receptor antagonist, LY341495 (1 mg/kg, i.p.). NAC still caused analgesia in mGlu3^−/−^ mice, but was inactive in mGlu2^−/−^ mice. In wild-type mice, NAC retained the analgesic activity in the formalin test when injected daily for 7 days, indicating the lack of tolerance. Both single and repeated injections of NAC also caused analgesia in the complete Freund’s adjuvant (CFA) model of chronic inflammatory pain, and, again, analgesia was abolished by LY341495. Data obtained in mice developing neuropathic pain in response to chronic constriction injury (CCI) of the sciatic nerve were divergent. In this model, a single injection of NAC caused analgesia that was reversed by LY341495, whereas repeated injections of NAC were ineffective. Thus, tolerance to NAC-induced analgesia developed in the CCI model, but not in models of inflammatory pain. The CFA and CCI models differed with respect to the expression levels of xCT (the catalytic subunit of Sx_c_^-^) and activator of G-protein signaling type-3 (AGS3) in the dorsal portion of the lumbar spinal cord. CFA-treated mice showed no change in either protein, whereas CCI mice showed an ipislateral reduction in xCT levels and a bilateral increase in AGS3 levels in the spinal cord.

**Conclusions:**

These data demonstrate that pharmacological activation of Sx_c_^-^ causes analgesia by reinforcing the endogenous activation of mGlu2 receptors. NAC has an excellent profile of safety and tolerability when clinically used as a mucolytic agent or in the management of acetaminophen overdose. Thus, our data encourage the use of NAC for the experimental treatment of inflammatory pain in humans.

## Background

Group-II metabotropic glutamate receptors (mGlu2 and mGlu3 receptors) regulate pain threshold and are considered as potential targets for novel analgesic drugs [1-3]. mGlu2/3 receptors regulate pain transmission at different levels of the pain neuraxis. In peripheral nociceptors, mGlu2/3 receptors inhibit pain transmission by restraining the activation of TRPV1 receptors and tetrodotoxin-resistant voltage-sensitive sodium channels [4-6]; in the dorsal horns of the spinal cord, mGlu2/3 receptors are presynaptically localized on primary afferent fibers, where they negatively modulate neurotransmitter release [7,8]. These receptors also regulate pain threshold in the periaqueductal grey and amygdala [9,10]. Recent findings suggest that analgesia caused by mGlu2/3 receptor agonists is primarily mediated by the activation of mGlu2 receptors [11]. However, mGlu3 receptors may also contribute to the regulation of pain threshold as suggested by the evidence that (i) mGlu3 receptors are up-regulated in the spinal cord and cerebral cortex in models of peripheral inflammation and hyperalgesia [12-14]; and (ii) the putative mGlu3 agonist, N-acetylaspartylglutamate (NAAG), or inhibitors of NAAG degradation cause analgesia in models of inflammatory or neuropathic pain [15-21]. Taken together, these data set the ground for the development of mGlu2/3 receptor agonists or mGlu2 receptor PAMs (positive allosteric modulators) as novel analgesic drugs [1-3]. However, a major pitfall associated with the use of mGlu2 receptor agonists for pain treatment is the development of tolerance, which occurs after only 4–5 days of repeated dosing [22,23]. One way to overcome this limitation is to enhance the expression of mGlu2 receptors. L-Acetylcarnitine (LAC) and histone deacetylase (HDAC) inhibitors cause analgesia without the development of tolerance by selectively increasing mGlu2 receptor expression in dorsal root ganglia (DRG) neurons and dorsal horns of the spinal cord. Both classes of drugs enhance transcription of the *Grm2* gene encoding mGlu2 receptor by acetylating p65/RelA, a member of the Nuclear Factor-κB (NFκB) family of transcription factors [1,23-25]. This finding contributes to explain the clinical efficacy of LAC in painful peripheral neuropathies [26-29] and holds promise for a potential use of HDAC inhibitors in the treatment of chronic pain. An alternative strategy is to reinforce the endogenous activation of mGlu2 receptors, considering that these receptors are mainly, albeit not exclusively, localized in the preterminal region of axon terminals and have limited access to synaptic glutamate [30]. The L-cystine/L-glutamate membrane exchanger (System x_c_^-^ or Sx_c_^-^), which mediates non-vesicular release of glutamate from astrocytes and microglia, represents a potential source for endogenous activation of presynaptic mGlu2/3 receptors [31]. Sx_c_^-^ is a membrane antiporter that mediates the chloride-dependent, sodium-independent, 1:1 exchange of extracellular L-cystine and intracellular L-glutamate, and provides the intracellular L-cysteine required for the synthesis of glutathione. Similarly to other members of heteromeric amino acid transporter family, Sx_c_^-^ is formed by a heavy chain 4F2hc subunit and a light chain xCT (SLC7A11) subunit. The xCT subunit mediates the transport of cystine and glutamate across the plasma membrane [32]. We hypothesized that drugs that promote Sx_c_^-^ activity, such as N-acetyl-cysteine (NAC) [32], could enhance the endogenous activation of mGlu2 receptors in the pain neuraxis, thereby producing analgesia.

Here, we report that systemic administration of NAC causes robust analgesia in mouse models of inflammatory and neuropathic pain, and that NAC acts by reinforcing the endogenous activation of mGlu2 receptors.

## Results

### Activation of Sx_c_^-^ by NAC causes analgesia in the second phase of the formalin test

**Figure 1:**
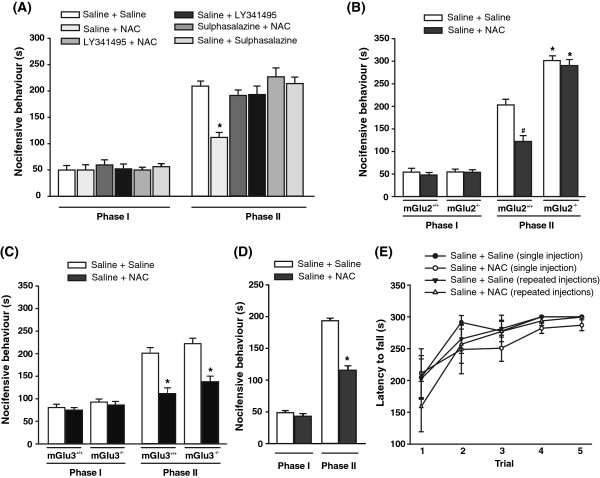
**Single and repeated injections of NAC cause analgesia in the phase II of the formalin test.** Nocifensive behavior of mice treated i.p. with a single injection of NAC (100 mg/kg) or saline preceded, 15 min earlier, by saline, sulphasalazine (8 mg/kg), or LY341495 (1 mg/kg) is shown in (**A**). Values are means ± SEM of 5–12 mice per group. p < 0.05 vs. all other groups of phase II (One-way ANOVA + Holm-Sidak’s test). Nocifensive behavior of mGlu2^−/−^ or mGlu3^−/−^ mice and their wild-type littermates treated i.p. with a single injection of NAC (100 mg/kg) or saline is shown in (**B**) and (**C**), respectively. Values are means ± SEM of 5 mice per group. p < 0.05 (Two-way ANOVA + Holm-Sidak’s test) vs. wild-type mice injected with saline alone (#) or vs. the corresponding groups of wild-type mice (*). Nocifensive behavior of normal mice treated i.p. with repeated injections of NAC (100 mg/kg) or saline, once a day for 7 days, is shown in (**D**). Values are means ± SEM of 6 mice per group. *p < 0.05 (Student’s t test) vs. phase II values of mice treated with saline. Motor performance of mice treated with single or repeated injections of NAC or saline on the accelerating rotarod is shown in (**E**). Values are means ± S.E.M. of 5 mice per group.

In a first set of experiments we assessed the analgesic activity of NAC in the formalin model of inflammatory pain. A single injection of NAC (100 mg/kg, i.p.; 30 min before the test) did not affect the first phase of nocifensive behavior, which reflects peripheral inflammatory pain. In contrast, NAC caused robust analgesia in the second phase of the formalin test (F_(5,37)_ = 25,63; p < 0.001), which involves mechanisms of central sensitization in the dorsal horns of the spinal cord [33,34] (Figure 1A). To study the involvement of Sx_c_^-^ in the action of NAC, we used the Sx_c_^-^ inhibitor sulphasalazine [35]. Because sulphasalazine is converted into the anti-inflammatory/analgesic drug, 5-aminosalycilic acid [35], we first tested different doses of sulphasalazine alone in the formalin test. Sulphasalazine caused analgesia at doses of 25, 50 or 100 mg/kg, i.p. (not shown), but not at doses <10 mg/kg, i.p. NAC-induced analgesia was abrogated by a 15-min pretreatment with 8 mg/kg of sulphasalazine (p < 0.001), or with the orthosteric mGlu2/3 receptor antagonist, LY341495 (1 mg/kg, i.p.) (p < 0.001) (Figure 1A). Neither sulphasalazine nor LY341495 influenced nocifensive behavior on their own (Figure 1A). The action of NAC was also examined in mGlu2^−/−^ mice and their wild-type littermates. As expected [11], mGlu2^−/−^ mice showed an enhanced nocifensive behavior in the second phase of the formalin test, as compared to wild-type mice (strain x treatment interaction: F_(1,16)_ = 12,96; p = 0.002) (Figure 1B). A single injection of NAC failed to cause analgesia in mGlu2^−/−^ mice (Figure 1B). We extended the study to mGlu3^−/−^ mice and their wild-type littermates from a CD1 genetic background. As opposed to data obtained in mGlu2^−/−^ mice, NAC fully retained its analgesic activity in mice lacking mGlu3 receptors (second phase of the formalin test: strain x treatment interaction: F_(1,16)_ = 35.738; p < 0.001) (Figure 1C).

To examine whether tolerance could develop to the analgesic activity of NAC, mice were treated with NAC (100 mg/kg, i.p.) once a day for 7 days, with the test being performed 30 min after the last injection. Repeated injections of NAC still caused analgesia in the second phase of the formalin test (t_(12)_ = 9,68; p < 0.001) (Figure 1D), suggesting the lack of tolerance.

To exclude that measurements of the analgesic activity of NAC could be biased by changes in motor activity we assessed motor coordination in mice receiving single or repeated injections of NAC using the rotarod test. Control mice were treated with saline i.p. once a day for 7 days. One group of mice received saline for 6 days followed by NAC (100 mg/kg, i.p.) on the 7^th^ day. Another group of mice was treated with NAC once a day for 7 days. Motor behavior was assessed for 5 consecutive trials (with intervals of 5 min), starting 30 min following the last injection. Motor performance was unaffected by single or repeated injections of NAC in the five trials (Figure 1E).

### NAC causes analgesia in the complete Freund’s adjuvant (CFA) model of chronic inflammatory pain via the activation of mGlu2 receptors

**Figure 2:**
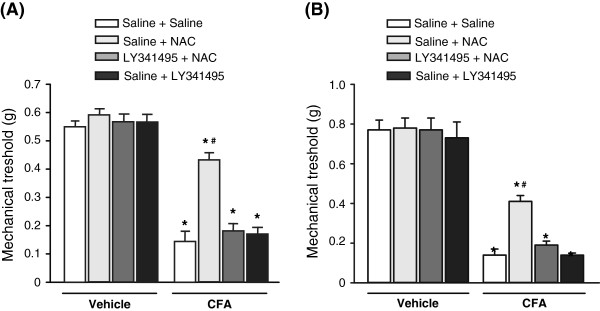
**Single and repeated injections of NAC cause analgesia in the CFA model of chronic inflammatory pain.** Mice were injected with vehicle (saline) or CFA in the right hind paw, and tested for mechanical pain thresholds after 7 days. In (**A**) mice were treated i.p. with a single injection of NAC (100 mg/kg) or saline preceded, 15 min earlier, by saline or LY341495 (1 mg/kg). In (**B**), mice were treated i.p. with saline or NAC for 7 days. Either saline or LY341495 (1 mg/kg) were injected i.p. 15 min prior to the last injection of NAC or saline. In both (A) and (B), values are means ± SEM of 9 mice per group. p < 0.05 (Two-way ANOVA + Holm-Sidak’s test) vs. the respective groups of control mice treated s.c. with vehicle (saline) in the hind paw (*), or vs. all other groups of CFA mice (#).

We extended the study to the CFA model of chronic inflammatory pain in mice receiving single or repeated injections of NAC. In acute experiments, NAC (100 mg/kg, i.p.) was administered only once, at day 7 following unilateral intraplantar injection of CFA. In chronic experiments, NAC was administered daily starting 4 h following CFA injection. Mechanical thresholds were measured after 7 days of treatment always 30 min following the last injection of NAC or saline. Both single and repeated injections of NAC caused a robust analgesia in the CFA model of chronic pain (CFA/vehicle x acute drug treatment: F_(3,64)_ = 3.46; p = 0.021; CFA/vehicle x repeated drug administrations: F_(3,64)_ = 3.77 p = 0.015) (Figure 2A,B). Again, the action of NAC was abrogated by LY341495 (1 mg/kg, i.p.), given 15 min before NAC in acute experiments, or 15 min before the last administration of NAC in chronic experiments (Figure 2A,B). Thus, a single injection of LY341495 was sufficient to abolish analgesia induced by repeated injections of NAC (Figure 2B).

### Single injection, but not repeated injections, of NAC causes analgesia in the chronic constriction injury (CCI) model of neuropathic pain

**Figure 3:**
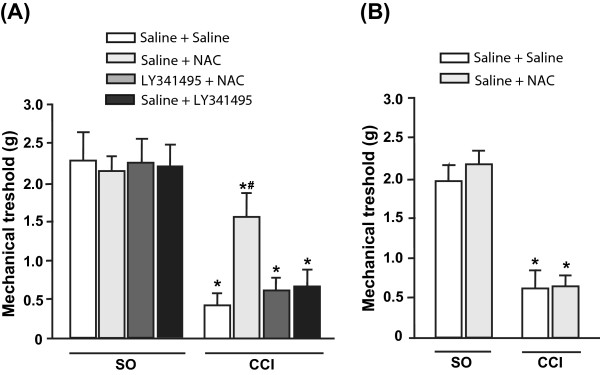
**Single injection of NAC causes analgesia in the CCI model of neuropathic pain.** (**A**), Mechanical allodynia analysis of CCI mice (14 days after surgery). In (**A**) mice were treated i.p. with a single injection of NAC (100 mg/kg) or saline preceded, 15 min earlier, by saline or LY341495 (1 mg/kg). In (**B**), mice were treated i.p. with saline or NAC for 7 days. Either saline or LY341495 (1 mg/kg) were injected i.p. 15 min prior to the last injection of NAC or saline. In both (A) and (B), values are means ± SEM of 11 mice per group. p < 0.05 (Two-way ANOVA + Holm-Sidak’s test) vs. the respective groups of sham operated (SO) mice (*), or vs. all other groups of CCI mice (#).

Mice were subjected to unilateral CCI of the sciatic nerve and tested for mechanical pain thresholds after 14 days. A single injection of NAC (100 mg/kg, i.p.) significantly increased pain thresholds in CCI mice (CCI/sham operated (SO) x drug treatment: F_(6,84)_ = 3.11 p < 0.001). Again, the action of NAC was abrogated by a 15-min pretreatment with LY341495 (1 mg/kg, i.p.), which was inactive on its own (Figure 3A). In the CCI model, repeated injections of NAC failed to induce analgesia (CCI/SO x drug treatment: F_(1,41)_ = 0.14; p > 0.05), suggesting that neuropathic pain facilitates the development of tolerance to the action of NAC (Figure 3B).

### The activation machinery of mGlu2 receptors is impaired in the CCI model of neuropathic pain but not in the CFA model of chronic inflammatory pain

**Figure 4:**
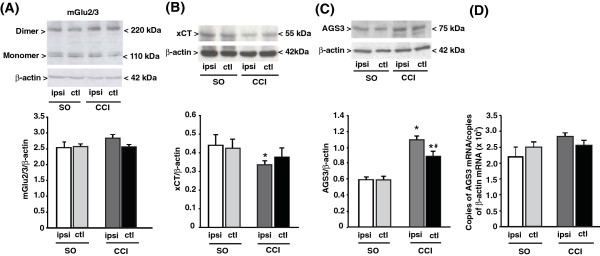
**Changes in xCT and AGS3 levels in the lumbar spinal cord of mice developing neuropathic pain.** Immunoblot analysis of mGlu2/3 receptors, xCT, and AGS3 in the ipsilateral (ipsi) and contralateral (ctl) dorsal portions of the lumbar spinal cord of sham operated animals (SO) and CCI mice is shown in (**A**), (**B**), and (**C**), respectively. AGS3 mRNA levels in the ipsilateral and contralateral DRG are shown in (**D**). Values are means ± S.E.M. of 6–8 individual determinations (from 2 groups of 8 mice). In (**B**), p < 0.05 (Two-way ANOVA + Holm-Sidak’s test) vs. the respective values of SO mice (*), or vs. values of the ipsilateral side of CCI mice (#).

**Figure 5:**
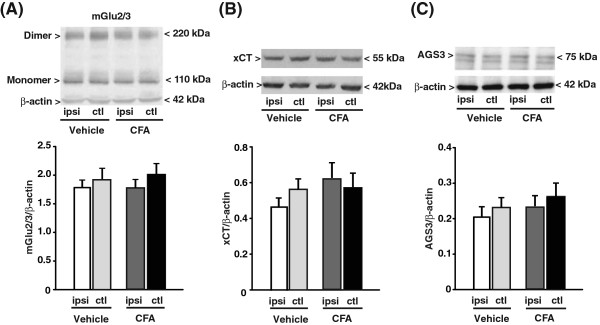
**Levels of mGlu2/3 receptors, xCT and AGS3 in the lumbar spinal cord of mice developing chronic inflammatory pain.** Same as in Figure 4, with the difference that mice were injected with vehicle (saline) or CFA in the hind paw. Values are means ± S.E.M of 6 mice per group.

Searching for mechanisms that could explain the refractoriness of CCI mice to repeated injections of NAC, we measured the levels of (i) mGlu2/3 receptors; (ii) the xCT catalytic subunit of the cystine/glutamate antiporter; and (iii) the Gi-protein regulator, type-3 activator of G-protein signaling (AGS3). Immunoblot analysis of mGlu2/3 receptors showed two bands at 100 kDa and >200 kDa corresponding to receptor monomers and dimers, respectively (see representative immunoblot in Figure 4A). In CCI mice, we found no changes in the expression of mGlu2/3 receptors in the contralateral and ipsilateral dorsal portions of the lumbar spinal cord after 14 days (Figure 4A). In contrast, the expression of xCT (detected as a single band at the expected molecular size of 55 kDa) was reduced ipsilaterally in the dorsal region of the lumbar spinal cord of CCI mice as compared to the ipsilateral region of SO mice (SO x CCI: F_(1,20)_ = 7.25 p = 0.014; CCI/SO x ipsilateral/contralateral: F_(1,20)_ = 0.47; p > =0.05) (Figure 4B). AGS3 (identified as a band at 75 kDa), which negatively regulates the Gα_i_-mediated signaling of mGlu2/3 receptors [21], was instead up-regulated both ipsilaterallly and contralaterally in CCI mice (CCI x SO: F_(1,28)_ = 1.046E + 002; p < 0.001; ipsilateral x contralateral: F_(1,28)_ = 9.78 p = 0.004; CCI/SO x ipsilateral/contralateral: F_(1,28)_ = 3,47 p > 0.05) (Figure 4C). The up-regulation of the AGS3 protein in the spinal cord was not associated with detectable changes in AGS3 mRNA levels in the DRG (the site of production of AGS3 present in the primary afferent fibers), although a trend to an increase was seen in the ipsilateral side (Figure 4D). Interestingly, levels of mGlu2/3 receptors, xCT, and AGS3 were unaltered in the spinal cord of CFA mice (Figure 5A-C).

## Discussion

Recent evidence highlights the role of glial Sx_c_^-^ in regulating the homeostasis of extracellular glutamate in the CNS, thereby contributing to physiological and pathological processes driven by ionotropic or metabotropic glutamate receptors. For example, Sx_c_^-^ -mediated efflux of glutamate from glioma cells provides a signal that allows tumor cell growth and migration *via* the activation of Ca^2+^-permeable AMPA receptors [36,37]. Peter Kalivas (University of South Caroline) and his colleagues have shown cocaine addiction in rats causes a down-regulation of Sx_c_^-^ and an up-regulation of AGS3, which both impair mGlu2/3 receptor signaling in the nucleus accumbens core. Treatment with NAC reverses cocaine-induced metaplasticity, and inhibits cocaine seeking [38-41]. Remarkably, NAC also reduces cocaine craving in humans [42], suggesting that the strategy to reinforce mGlu2/3 signaling can be applied to human pathology.

Here, NAC caused robust analgesia in models of inflammatory and neuropathic pain, and its action was sensitive to mGlu2/3 receptor blockade. In the formalin model, NAC did not cause analgesia in mice lacking mGlu2 receptors, but retained its activity in mice lacking mGlu3 receptors. Thus, at least in the formalin model, the action of NAC appeared to be mediated by mGlu2 receptors. These findings are intriguing because mGlu3 receptors also contribute to the regulation of pain transmission (see Introduction and references therein), and are expected to be activated by the glutamate released from astrocytes *via* the cystine/glutamate antiporter. One possible explanation is that the mGlu3 receptor, which has a high affinity for glutamate, is saturated by the ambient glutamate and cannot be further activated by drug that enhances glutamate release from astrocytes. However, the evidence that nocifensive behavior was not amplified in mGlu3^−/−^ mice (as opposed to mGlu2^−/−^ mice) (see also ref. 11) calls into question the role of mGlu3 receptors in the endogenous regulation of pain transmission. Another interesting finding was that NAC reduced nocifensive behavior exclusively in the second phase of the formalin test, whereas mGlu2/3 receptor agonists also cause analgesia in the first phase of the test, providing that the mGlu2 receptor is present [11]. We speculate that the action of mGlu2/3 receptor agonists in the first phase of the formalin test is mainly mediated by mGlu2 receptors expressed on peripheral nociceptors [4,43-45], which may not be targeted by the glutamate released *via* the glutamate/cystine antiporter. The second phase of the formalin test reflects mechanisms of nociceptive sensitization in the dorsal horns of the spinal cord [33,34], where mGlu2 receptors are likely to be activated by the glutamate released from astrocytes. To demonstrate that NAC caused analgesia by increasing Sx_c_^-^ activity, we used the drug sulphasalazine in the formalin model. The pharmacology of Sx_c_^-^ is nicely reviewed by Bridges et al. [32]. (S)-4-Carboxyphenylglycine (4-S-CPG), (S)-4-carboxy-3-hydroxyphenylglycine (4C3HPG) [46,47] and sulphasalazine [35] are among the most potent inhibitors of Sx_c_^-^. We could not use 4-S-CPG or 4C3HPG because these drugs can also activate mGlu2/3 receptors [48]. Sulphasalzine is a pro-drug containing sulphapyridine and 5-amino salicylic acid, which is widely used in the clinic for the oral or rectal treatment of inflammatory bowel disorders [49]. Because significant amounts of 5-amino salicylic acid can be released in response to i.p. injection of sulphasalazine, we first searched for a dose of sulphasalazine that was not analgesic *per se*. At the dose of 8 mg/kg, sulphasalazine did not influence nocifensive behavior on its own, but abrogated the analgesic activity of NAC indicating that NAC acted by stimulating Sx_c_^-^ activity.

An intriguing finding was that no tolerance to NAC-induced analgesia developed in models of inflammatory pain, whereas repeated administrations of NAC failed to induce analgesia in the CCI model of neuropathic pain. We searched for molecular events that could have limited analgesia in response to repeated injections of NAC by measuring the levels of mGlu2/3 receptors, xCT and AGS3 in the ipsi- and contralateral dorsal regions of the spinal cord of CFA and CCI mice. Interestingly, CFA mice did not show changes in the levels of any of these proteins, whereas CCI mice showed a reduced expression of xCT ipsilaterally, and an increased expression of AGS3 bilaterally. AGS3 is a member of a family of G-protein regulators that influence nucleotide exchange at the α subunit of Gi/o proteins [50]. AGS3 stabilizes Gα_i_ in the GDP-bound inactive configuration, thereby inhibiting Gα_i_-mediated transduction mechanisms in spite of its name (AGS stands for “activator of G-protein signaling”) [50]. mGlu2/3 receptors are coupled to Gi/Go proteins, and their activation negatively modulates adenylyl cyclase activity [30]. It is possible that, in the CCI model, unidentified adaptation responses to NAC developed in a context of neuroplastic changes limiting the endogenous activation of mGlu2 receptors (i.e., a reduced expression of xCT and an increased expression of AGS3). This may cause refractoriness to repeated injections of NAC. In contrast, adaptation responses to NAC would not be sufficient to cause tolerance in the CFA model of inflammatory pain, in which the endogenous machinery of mGlu2 receptor activation was apparently intact. Further studies are needed to identify the precise mechanism by which tolerance to NAC develops in models of neuropathic pain, but not in models of inflammatory pain.

## Conclusions

In conclusion, these data demonstrate that the strategy of reinforcing the endogenous activation of mGlu2 receptors can be successfully applied to the treatment of pain. NAC is widely used in the clinic as a mucolytic agent, as well as in the treatment of acetaminophen poisoning and contrast-medium-induced nephropathy [51-54]. The excellent safety profile of NAC makes this drug a novel potential candidate for the experimental treatment of chronic inflammatory pain in humans.

## Methods

### Drugs

NAC, *aka* 6-oxo-3-(2-[4-(*N*-pyridin-2-ylsulphamoyl)phenyl]diazenyl)cyclohexa-1,4-dienecarboxylic acid (sulphasalazine), and formaldehyde were purchased from Sigma Aldrich (Milano, Italy). (2*S*)-2-Amino-2-[(1*S*,2*S*)-2-carboxycycloprop-1-yl]-3-(xanth-9-yl) propanoic acid (LY341495) was purchased from Tocris Cookson (Avonmouth, Bristol, UK).

### Animals

All experiments were carried out according to the European (86/609/EEC) and Italian (D: Lgs. 116/92) guidelines of animal care. The experimental protocol was approved by the Italian Ministry of Health. All efforts were made to minimize animal suffering and the number of animals used. In most of the experiments, we used adult male C57BL/6 J mice (20–25 g, b.w.) purchased from Charles River (Calco, Italy). We also used mGlu2 and mGlu3 receptor knockout mice (mGlu2^−/−^ and mGlu3^−/−^ mice). mGlu2^−/−^ mice from a C57BL/6 J genetic background were generated in the laboratory of Prof. Shigetada Nakanishi at Kyoto University, Japan. Knockout mice were backcrossed with C57BL/6 J wild-type mice for the generation of mGlu2^+/−^ mice. mGlu3^−/−^ mice from a CD1 genetic background were kindly provided by Eli Lilly and Company (IN, USA). Knockout mice were backcrossed with CD1 wild-type mice for the generation of mGlu3^+/−^ mice. All mice were individually genotyped for the mGlu2 and mGlu3 receptor gene by PCR. Knockout mice and their wild-type littermates generated by heterozygous crosses were used for the assessment of nocifensive behavior in the formalin test. All mice were housed 5 for cages, under a standard 12/12 h light/dark cycle with food and water *ad libitum*.

#### Formalin test

Acute inflammatory pain was assessed using the formalin test. Ten μl of a formalin solution (2% for C57BL/6 J mice and 5% for CD1 mice) was injected s.c. into the plantar surface of the right hind paw. After injection, mice were immediately placed in a plexiglass box (20 × 15 × 15 cm) surrounded by mirrors to allow the observation of nociceptive responses that include licking, lifting and shaking of the injected paw. Tests were performed between 08:00 h and 12:00 h to minimize variability. After formalin injection, mice were observed for 1 h and their behaviors were recorded by researchers blind to genotypes and drug treatments. Formalin scores were separated into two phases, phase I (0–10 min) and phase II (20–60 min). The mean behavioral score was calculated in blocks of 5 min for each of the two phases. A mean response was then calculated for each phase [11]. In acute experiments, 6 groups of mice (n = 5–12 per group) were treated i.p., as follows: (i) one injection of saline followed by another injection of saline; (ii) one injection of NAC (100 mg/kg) followed by one injection of saline; (iii) one injection of LY341495 (1 mg/kg) followed by one injection of saline; (iv) one injection of sulphalazine (8 mg/kg) followed by one injection of saline; (v) one injection of LY341495 followed by one injection of NAC; and (vi) one injection of sulphalazine followed by one injection of NAC. In all groups the two injections were performed with 15 min of interval. Formalin was injected in the hind paw 30 min after the second i.p. injection. In chronic experiments, 2 groups of 6 mice were treated daily i.p. with saline or NAC (100 mg/kg) for 7 days. Formalin was injected 30 min after the last injection of saline or NAC. Two additional groups of mGlu2^−/−^ mice and two groups of wild-type littermates (n = 5 per group) received a single i.p. injection of saline or NAC (100 mg/kg) 30 min prior to formalin injection.

#### The CFA model of chronic inflammatory pain

Tissue inflammation was induced by s.c. injections of 20 μl of Complete Freund’s Adjuvant (CFA, F5881 Sigma-Aldrich; 1 mg/ml) in the dorsal surface of the right hind paw; control mice were injected s.c. with saline in the right hind paw. Mechanical pain thresholds were assessed with von Frey filaments (see below) 7 days after injection of CFA or vehicle (saline) in the hind paw. For acute experiments, 4 groups of control and 4 groups of CFA-injected mice (n = 9 mice per group) were treated i.p. as follows: (i) one injection of saline followed by another injection of saline; (ii) one injection of NAC (100 mg/kg) followed by one injection of saline; (iii) one injection of LY341495 (1 mg/kg) followed by one injection of saline; (iv) one injection of LY341495 followed by one injection of NAC. The two i.p. injections were carried out with 15 min of interval. All treatments were performed at day 7 after vehicle or CFA injections in the hind paw, with the second i.p. injection being performed 30 min prior to the assessment of mechanical pain thresholds. For chronic treatments, 4 groups of control and 4 groups of CFA-injected mice (n = 9 per group) were treated using the same protocol of acute experiments with the difference that drugs were injected daily for 7 days starting 10 min following CFA (or saline) injection. Mechanical pain thresholds were measured 30 min after the last i.p. injection. One additional group of control mice and one additional group of CFA-injected mice (n = 6 per group) were used for biochemical experiments. These mice were tested for mechanical pain thresholds at day 7, and killed after 6 h.

#### Induction of chronic constriction injury (CCI) of the sciatic nerve

Seventy-four mice subjected to CCI of the sciatic nerve, and 74 sham operated (SO) mice were used for pharmacological experiments. All selected CCI mice did not display gross deficits in motor behavior that might have influenced the assessment of mechanical allodynia. Chronic constriction of the sciatic nerve was carried out under pentobarbital anesthesia (50 mg/kg, i.p.), as described by Bennett and Xie [55]. In brief, the biceps femoris and the gluteus superficialis were separated by blunt dissection, and the left sciatic nerve was exposed. CCI was produced by tying two ligatures around the sciatic nerve. The ligatures were tied loosely around the nerve with 1 mm spacing, until they elicited a brief twitch in the respective hind limb, which prevented over-tightening of the ligations, taking care to preserve epineural circulation. The incision was cleaned and the skin was closed with 2–3 ligatures of 5–0 dermalon. Mice were then placed on a warmed surface and, following recovery, they were returned to their home cages and checked routinely for 72 h. In SO mice, the left sciatic nerve was exposed without ligature. Mechanical allodynia (see below) was assessed 14 days after surgery. The development of mechanical allodynia was evaluated by using the von Frey filaments. All animals were tested before surgery and then 14 days after surgery. Six groups of CCI mice and 4 groups of SO mice (n = 11 per group) were used for pharmacological experiments with NAC and/or LY341495. One group of CCI mice and one group of SO mice (n = 8 per group) were used for biochemical analysis. For pharmacological experiments, the following groups of SO or CCI mice were treated i.p. as follows. Acute experiments: (i) one injection of saline followed by another injection of saline; (ii) one injection of saline followed by one injection of NAC (100 mg/kg); (iii) one injection of LY341495 (1 mg/kg) followed by one injection of saline; and (iv) one injection of LY341495 followed by one injection of NAC. The two i.p. injections were separated by a 15-min interval. Chronic experiments: (i) daily injections with saline for 7 days; and (ii) daily injections with NAC (100 mg/kg) for 7 days. In acute experiments, pain thresholds were measured 1 h after the last injection. In chronic experiments, treatment started on day 7 after surgery, and pain thresholds were measured 1 h after the last injection. For biochemical experiments, one additional group of SO and one additional group of CCI mice (n = 8 per group) were tested for mechanical thresholds at 14 days, and killed after 6 h.

### Assessment of mechanical allodynia

Mechanical allodynia was quantified by measuring the hind paw withdrawal response to von Frey filament stimulation. In brief, animals were placed in a Plexiglas® box (20 cm high, 9 cm diameter) with a wire grid bottom through which the von Frey filaments (North Coast Medical, Inc., San Jose, CA, USA) bending force range from 0.008 to 3.5 g, were applied by using a modified version of the up-down paradigm, as previously reported by Chaplan [56]. The filaments were applied 5 times each, in order of increasing forces, and pressed perpendicularly to the plantar surface of the hindpaw until they bent. The first filament which evoked at least 3 responses was assigned as the pain threshold in grams. For the assessment of mechanical sensitivity, mice were placed in individual cages with elevated mesh floor 1 h before all behavioral testing. Mechanical allodynia was determined with a set of calibrated von Frey filaments used to stimulate the dorsal side of both right and left paws.

### Assessment of motor performance by the rotarod test

The rotarod apparatus consisted of a rotating horizontal cylinder (30 mm) and a motor driver control unit (Ugo Basile, Varese, Italy). The cylinder was divided into five separate rotating compartments and fully enclosed to ensure that the mice did not jump out of their area. Mice were placed on the rod, which was rotating at an accelerating speed from 4 to 40 rpm in 5 min. The time the mice remained on the rod was automatically recorded by timers. Five mice for each group were treated with single or repeated i.p. injection of saline and NAC (100 mg/kg). Thirty minutes later mice were placed on the rotarod apparatus and five consecutive acceleration trials were carried out with an interval of five min between each trial.

### Biochemical experiments

#### Western blot analysis

The dorsal portions of the lumbar spinal cord containing the dorsal horns were dissected out and homogenized at 4 °C in Tris–HCl buffer, containing 1 mM PMSF, pH 7.4, and an aliquot was used for protein determinations. Equal amounts of proteins (30 μg) from supernatants were separated by 8% SDS polyacrilamide gel for the detection of mGlu2/3 receptor, while for the detection of xCT and AGS3 proteins was used 10% SDS polyacrilamide gel. After separation, proteins were transferred on immuno-blot PVDF membranes. Filters were blocked for 2 h at 4 °C in TTBS buffer containing 5% non-fat dry milk. mGluR2/3 was labeled using anti-Glutamate Receptor 2/3, Metabotropic (mGluR2/3) produced in rabbit (1:1000, Sigma) and secondary anti-rabbit antibody (peroxidase-coupled antirabbit 1:7.000 Calbiochem, Milano, Italy). Blots for xCT were incubated overnight at 4 °C with the respective antibodies: anti Mouse Cystine/Glutamic Acid Trasporter (xc^-^), (1:1.500, rabbit polyclonal, Transgenic, Tokyo, Japan). Filters were washed three times with TTBS buffer and then incubated for 1 h with secondary antibodies (peroxidase-coupled anti-rabbit 1:7.000 Calbiochem, Milano, Italy). Blots for AGS3 were incubated with the goat polyclonal antibody for AGS3 (1:500, AGS3 (A-20): sc-1639, Santa Cruz Biotechnology, Inc. CA, USA). Filters were washed three times with TTBS buffer and then incubated for 1 h with secondary antibodies (peroxidase-coupled donkey anti-goat 1:5.000, Santa Cruz Biotechnology, Inc., Temecula, CA, USA). Immunostaining was revealed by enhanced-chemiluminescence luminosity (Amersham Pharmacia Biotech, Arlington Height, IL). The blots were reprobed with anti-β-actin monoclonal antibody (1:250, Sigma, St. Louis, MO).

#### Real-time RT-PCR

DRGs were dissected bilaterally, and total RNA was isolated using the TRIzol reagent (Invitrogen) according to the manufacturer’s protocol and retrotranscribed into cDNA by using SuperScript III Reverse Transcriptase (Invitrogen). Real-Time RT-PCR was performed on the Step One Plus Applied Biosystems. PCR was performed by using Power SYBR Green PCR Master Mix Kit (Applied Biosystems) according to the manufacturer’s instructions. Thermal cycler conditions were as follows: 5 min at 50 °C, 2 min at 95 °C, 40 cycles of denaturation (45 s at 95 °C), and combined annealing/extension (1 min at 60 °C). The sequences of GAPDH and AGS3 primers used were: GAPDH forward 5′-CGTCCCGTAGACAAAATGGT-3′ and reverse 5′- TCAATGAAGGGGTCGTTGAT-3′; AGS3 forward 5′-TGCGGCACCTAGTCATTGC-3′ and 5′- TGTCAGTTCTCCGTTTCGGTC-3′. Concentrations of mRNA were calculated from serially diluted standard curves simultaneously amplified with the samples and normalized with respect to GAPDH mRNA levels.

### Statistical analysis

Statistical analysis was carried out by using the Student’s t test (Figure 1D); One-Way ANOVA (Figure 1A) or Two-Way ANOVA (all other Figures) followed by Holm-Sidak’s test. *p* values <0.05 were considered significant.

## Abbreviations

mGlu: Metabotropic glutamate receptor; System x_c_^-^ or Sx_c_^-^: Cystine/glutamate antiporter; NAC: N-acetyl-cysteine; CFA: Complete Freund’s adjuvant; CCI: Chronic constriction injury; AGS3: Activator of the G-protein signaling, type-3; xCT: Catalytic subunit of Sx_c_^-^; PAMs: Positive allosteric modulators; LAC: L-Acetylcarnitine; HDAC: Histone deacetylase; DRG: Dorsal Root Ganglia.

## Competing interests

The author(s) declare that they have no competing interests.

## Authors’ contributions

MB, SN, CZ and FF designed and performed *in vivo* experiments and analysed data. MC and MM performed real-time PCR experiments. GB, VB and RG contributed to experiment design and supervised research. FN designed experiments, supervised research and wrote the manuscript. All authors read and approved the final manuscript.
